# Burnout and depression among psychiatry residents during COVID-19 pandemic

**DOI:** 10.1186/s12960-021-00584-1

**Published:** 2021-04-06

**Authors:** Abdulmajeed A. Alkhamees, Hatem Assiri, Hatim Yousef Alharbi, Abdullah Nasser, Mohammad A. Alkhamees

**Affiliations:** 1 Department of Medicine, Unaizah College of Medicine and Medical Sciences, Qassim University, Buraydah, Al Qassim Saudi Arabia; 2 Adult Mental Health Department At King Abdelaziz Medical City, Ministry of National Guard, Health Affairs-Riyadh, Riyadh, Saudi Arabia; 3 Department of Psychiatry, College of Medicine, Qassim University, Buraydah, Saudi Arabia; 4 Department of Urology, College of Medicine, Majmaah University, Al Majma’ah, Saudi Arabia

**Keywords:** COVID 19, Burnout, Depression, Psychiatry, Resident, Pandemic, Saudi Arabia

## Abstract

Very few studies have been concerned with assessing the prevalence of burnout and depressive symptoms, especially during an infectious outbreak on non-frontline health care workers, such as a psychiatrist. In such instances, the role of psychiatrists and other mental health providers as a source of psychological support to the public and frontline workers is indispensable and valuable. This study aims to assess the prevalence of burnout and depressive symptoms, and their correlation, during the COVID-19 pandemic among psychiatry residents in Saudi Arabia. A total of 121 out of 150 psychiatry residents in Saudi Arabia completed the Maslach Burnout Inventory and Patient’s Health Questionnaire for the assessment of burnout and depressive symptoms. Burnout symptoms were found in 27.3%, and another 27.3% reported having depression symptoms. In addition, 16.5% reported having both burnout and depressive symptoms, with a significant relationship between them. Participants in the first 2 years of training and having a history of receiving mental health treatment in the past 2 years were at higher risk. The need is urgent to increase investment in mental health services and to construct a plan to reduce this risk of burnout and depression among psychiatrists by developing preventative strategies to prevent burnout and promote wellness is more important than ever.

## Introduction

The coronavirus disease of 2019 (COVID-19) pandemic has left health-care systems around the globe faced with challenging difficulties combating the pandemic and its impact. Health care providers have responded tirelessly to deal with these difficult times. The pandemic has left health care providers facing increased work demands and related stress, which has worsened already-existing burnout among health care providers [1].

After the first case was discovered in Saudi Arabia, the government response was immediate and swift; they launched a social media campaign encouraging people to stay at home and to practice social distancing, and to follow the instruction put out by the Ministry of Health [2]. On March 23, a lockdown was imposed on Mecca, Medina, and Riyadh with travel restrictions all over the country, and within the next 10 days, the curfew was extended to 24 h [3].

The notion of burnout was introduced in the scientific literature by Herbert J. Freudenberger’s work in 1974 [4]. Since then, this concept has evolved, and various definitions have emerged. Maslach and Jackson promoted the most commonly used definition [5]. They defined burnout as a psychological syndrome affecting mainly professionals working with other people in difficult circumstances, such as health care providers. Burnout is characterized by emotional exhaustion (tiredness and a feeling of being unable to provide more to others), depersonalization (impersonal feelings toward their patients, dealing with them as objects), and deficiency in the feeling of personal achievements [6].

The idea of overlap between burnout and depression has been supported by many published papers, including the results of research conducted among health care workers, which proposed that burnout is a manifestation of the depressive syndrome [7], 8.

Furthermore, Ahola et al. concluded that using a person-centered approach, depressive symptoms and burnout appear to form a single entity together or to develop in a parallel manner. These findings accentuate the conceptual similarity of burnout and depressive symptoms in a work context [9].

Numerous studies have explored the effect of an infectious disease outbreak on health care workers’ mental health. Recently, Ricci-Cabello et al. conducted a rapid systematic review that included 56 studies. They assessed a wide range of disturbing psychological reactions for their impact on the mental health of workers providing health care during or after health emergencies caused by viral epidemic outbreaks. Anxiety (45%) was the highest pooled prevalence, followed by depression (38%), acute stress disorder (31%), and post-traumatic stress disorder (19%), while the prevalence of burnout was 29% (in 1,168 participants), as reported by three studies [10].

In 2016, Jovanovic conducted an online survey of burnout syndrome among psychiatric trainees in 22 countries and found that about 36.7% had severe burnout [11].

In addition to the stressors of being a health-care provider, being a psychiatry trainee added more and different burdens, such as perceived stigma related to their chosen specialty, the ongoing demands of establishing therapeutic relationships with their patients, personal threats from violent patients, and patient suicide [12].

Tackling the mental health of health care workers during this pandemic is essential and will strengthen the capacity of health-care systems [13]. Unfortunately, few studies have assessed the prevalence of burnout during pandemic globally and locally. In addition, there is a lack of knowledge about the impact of an infectious disease outbreak on non-frontline health care workers such as psychiatrists. The role of psychiatrists and other mental health providers in such an instance as a source of psychological support and reassurance to the public and frontline workers is indispensable and valuable.

Furthermore, many authors tried to assess common sociodemographic factors that correlate with experiencing burnout and depressive symptoms among psychiatry residents. Tracking and identifying these factors are of great importance, as it allows us from a preventive perspective to identify and provide the necessary interventions early and for those who are more vulnerable than the other to the occurrence of burnout and depression. In their systematic review, Min Kai Chan et al. [14], in their systematic review found that having children being in early years of training (junior level) associated with reporting more burnout. Regarding depression being female and junior level of training were related to increase risk of developing depressive symptoms compared to counterpart [15].

To the best of our knowledge, there is a lack of studies that try to explore the relationship between these sociodemographic characteristics and the rates of burnout and depression on mental health trainees, especially during the period of pandemics.

This study aims to assess the prevalence of burnout and depression, and their correlation, during the COVID-19 pandemic among psychiatry residents in Saudi Arabia. A secondary aim is to assess sociodemographic characteristics and their correlation with burnout and depression among psychiatry residents in Saudi Arabia.

## Materials and methods

### Study design and setting

This study followed a cross-sectional design to assess psychiatry resident burnout and depression symptoms during the COVID-19 pandemic after the lockdown was imposed in Saudi Arabia. There are more than 150 psychiatry residents in Saudi Arabia divided into three training locations, the program consists of four training years divided into a junior level (R1–R2) and a senior level (R2–R3) with rotation in inpatient psychiatry unit and outpatient clinic and rotation in consultation–liaison psychiatry and other different rotations, and residents are required to pass a yearly promotional exam to pass to the next year.

All Saudi psychiatry residents located in Saudi Arabia were included. We used an online questionnaire distributed to all residents using WhatsApp groups. Physical distribution was not feasible due to the lockdown in the Kingdom at that time.

### Study procedure

The survey was distributed after the curfew was imposed in Saudi Arabia, and the practice of social distancing was encouraged. The Saudi Ministry of Health enforced a strict protocol to reduce unnecessary hospital visits. We followed an online data collection technique. The survey was done online using a common platform, google survey (Google LLC, Mountain View, California, USA). The study protocol was approved by the Institutional Review Board of Qassim University (No.19-10-02). All participants were informed about the study purposes and provided informed consent. Data were kept confidential and were not disclosed unless for study purposes. Data were collected over a 1-month period (March 15 to April 23, 2020).

### Variables and instruments

The survey included sociodemographic data—participants’ gender, age, marital status, parental status—as well as the location and level of the residency program. Participants also completed the Maslach Burnout Inventory (Human Services Survey; MBI-HSS) and the Patient’s Health Questionnaire (PHQ-9) for the assessment of burnout and depression.

The MBI-HSS is an assessment test developed by Maslach and Jackson in 1981 to measure personally perceived burnout [16, 17]. This scale has been used extensively to measure burnout in medical team members. The scale includes three domains: 1) emotional exhaustion (EE), 2) depersonalization (DP), and 3) personal accomplishment (PA). Each item measures a specific domain based on a 7-point scale, varying from “never” to “every day.” Scores for EE, DP, and PA range from 0 to 54, 0 to 30, and 0 to 48, respectively. [18]. High scores on the EE (≥ 27) and DP (≥ 13) subscales or a low score on the PA subscale (≤ 31) were considered highly suggestive of burnout symptoms [18]. Respondents with scores ≥ 27 on the EE subscale and/or ≥ 13 on the DP subscale were considered to be suffering from burnout [16, 17].

The PHQ-9 is a self-administered diagnostic instrument used for identifying depressive symptoms and monitoring their severity. The PHQ-9 has been validated in several studies from different populations [19]. Total scores of 10–14 points, 15–19 points, and 20–27 points indicate, respectively, moderate, moderately severe, and severe levels of depressive symptoms. Participant with a score of 10 or more on the PHQ-9 was considered as having depressive symptoms. [20], 21.

Both the MBI and PHQ-9 were found to have high internal consistency, with the 22-item MBI having a Cronbach’s alpha of 0.80 and the 9-item PHQ-9 having a Cronbach’s alpha of 0.89. When tested for their reliability, each of the subscales of the MBI performed well, with the Cronbach’s alpha of the EE, DP, and PA subscales being 0.91, 0.81, and 0.72, respectively.

### Statistical analysis

In line with the data collection approach earlier described, data was collected from respondents and responses entered into a Microsoft Excel spreadsheet and subsequently transferred to IBM SPSS Statistics for Windows, version 22.0 (IBM Corp., Armonk, N.Y., USA) for analysis. Descriptive analysis was carried out and the results presented as percentages and frequencies for categorical variables; and means and standard deviation for continuous variables. Univariate test of associations was carried out to determine associations between categorical variables and outcomes such as burnout and depression. Logistic regression was carried out to evaluate independent associations between the outcomes (burnout/depression) and factors such as sociodemographic and educational characteristics. The results of the univariate tests and logistic regressions were presented using crude odds ratio (COR) and adjusted odds ratio (AOR) with 95% confidence interval (CI). All tests were carried out at a significance level set at p < 0.05. Adjusted ORs for the relationship between depression and burnout were based on adjustment for components of the burnout scale. Similarly, adjusted ORs for the relationship between sociodemographic/educational factors and burnout/depression were based on adjustment for potential confounders such as "age", "sex", "marital status", "raising children", "current level", and "got mental help".

## Results

### Sociodemographic and educational characteristics

A total of 121 out of 150 psychiatry residents responded (response rate 80.6%), as presented in Table 1. The majority of them were within the 24–28-year age range (67.8%), closely followed by those between the ages of 29 and 33 years (31.4%). There were more males (57.9%) than females. With regard to their marital statuses, 52.1% of them were single, while another 44.6% were married at the time of the study. Only about a quarter of the study population had children they were currently raising (24.0%) at the time data was collected. For their psychiatry residency programs, most of the respondents were having theirs in Riyadh (42.1%), followed by those in Jeddah (32.2%) and Dammam (25.6%). The split across the four levels of psychiatry residency (R1–R4) is similar to the largest groups being those in R2 (27.3%) and R3 (26.4%). About a quarter of the study population (22.3%) had received mental health help for different reasons within the previous 2 years.

**Table 1:** Sociodemographic and educational characteristics of the study population (n = 121)

Variables	Frequency	Percent
Age		
24–28 years	82	67.8
29–33 years	38	31.4
34–38 years	1	0.8
Gender		
Male	70	57.9
Female	51	42.1
Marital status		
Single	63	52.1
Married	54	44.6
Divorced	4	3.3
Raising children		
No	92	76.0
Yes	29	24.0
Location of psychiatry residency program		
Riyadh	51	42.1
Dammam	31	25.6
Jeddah	39	32.2
Current level in residency		
R1	27	22.3
R2	33	27.3
R3	32	26.4
R4	29	24.0
Received mental help in the last 2 years	33	27.3
Yes	27	22.3
No	94	77.7

### Prevalence of burnout and depressive symptoms among the study population

The summary of findings on the prevalence of burnout and depressive symptoms is presented in Table 2. Overall, 26.4% of the entire population had high emotional exhaustion, 10.7% met the criteria for high depersonalization, and 24.0% demonstrated low personal accomplishment. Combining these findings, about a quarter of the population had either high emotional exhaustion and/or high depersonalization and were determined to be suffering from burnout (27.3%). Going further to look at depressive symptoms, 27.3% of the population were determined to suffer from depressive symptoms, with a split into those who had moderate depressive symptoms in the majority (60.6%), followed by those with moderately severe depressive symptoms (21.2%), and severe depressive symptoms (18.2%).

**Table 2:** Prevalence of burnout and depressive symptoms among the study population (n = 121)

Variables	Frequency	Percent (%)
Burnout syndrome^a^ *(n* = *121)*		
Yes	33	27.3
No	88	72.7
Burnout subscales *(n* = *121)*		
High emotional exhaustion	32	26.4
High depersonalization	13	10.7
Low personal accomplishment	29	24.0
Depressive symptoms (*n* = *121)*		
Yes	33	27.3
No	88	72.7
Severity of depressive symptoms (*n* = *33)*		
Moderate depressive symptoms	20	60.6
Moderately severe depressive symptoms	7	21.2
Severe depressive symptoms	6	18.2

^a^Determined based on respondents having a high score on the emotional exhaustion and/or depersonalization subscales (see “Methods”)

### Relationship between burnout and depressive symptoms

**Fig. 1:**
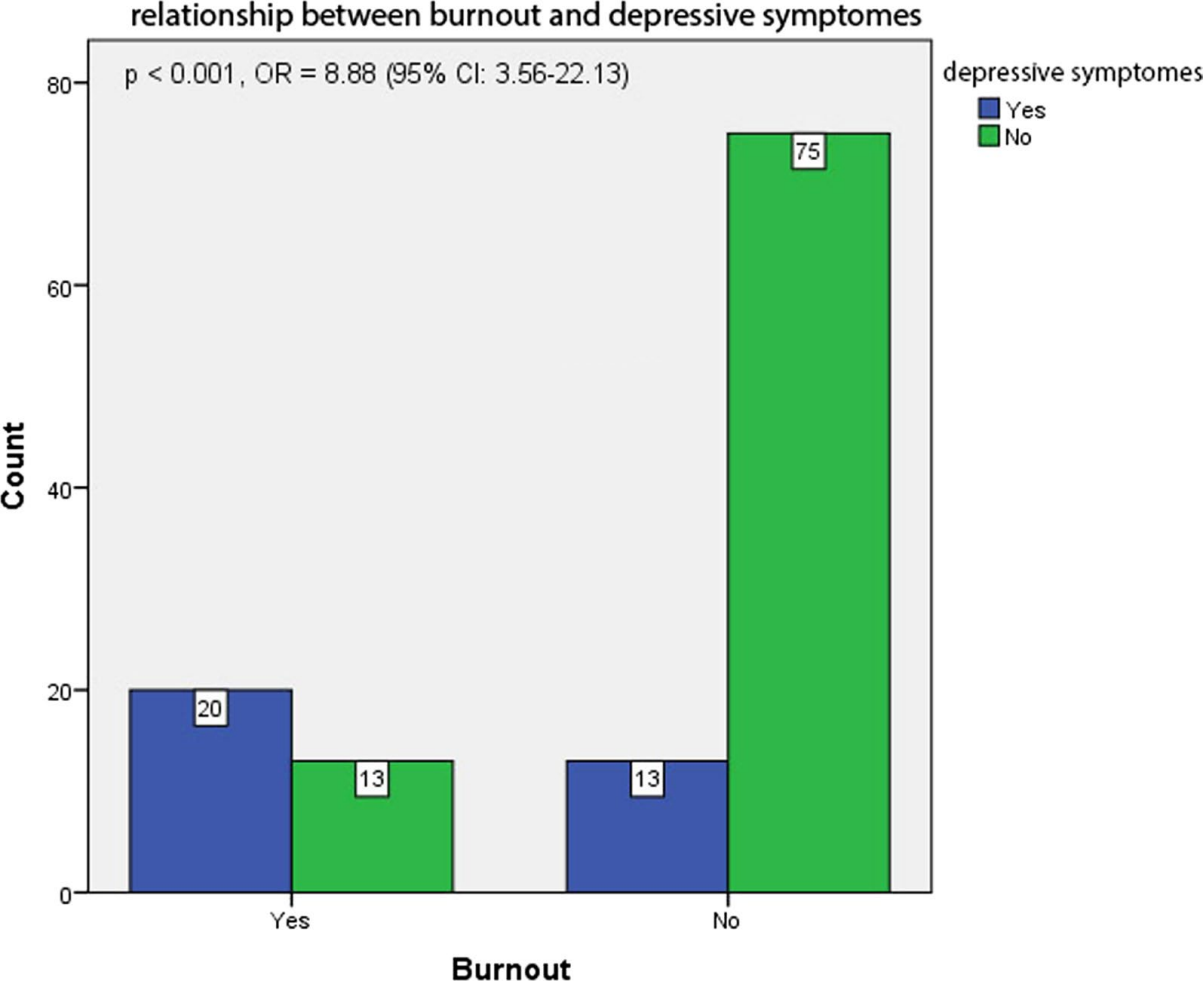
Relationship between burnout and depressive symptoms among the study population (Chi square = 25.42, *p* < 0.001, COR: 8.88, 95% CI 3.56–22.13)

There was a relationship between burnout and depressive symptoms, as illustrated in Fig. 1. Respondents who suffered from burnout were significantly 8.88 times more likely to have depressive symptoms (95% CI 3.56–22.13, p < 0.001). The relationship between respondents’ performance on the burnout scales and depressive symptoms are summarized in Table 3. High scores on all three subscales (emotional exhaustion, depersonalization, and personal accomplishment) were significantly predictive of depressive symptoms (p < 0.05).

**Table 3:** Relationship between burnout subscales and depressive symptoms (n = 121)

Subscales	Values	Depressive symptoms		Total	Crude OR		Adjusted OR	
		Yes (n = 33)	No (n = 88)		OR	95% CI	OR	95% CI
High emotional exhaustion	Yes	20 (62.5%)	12 (37.5%)	32	9.74*	3.86–24.61	5.60*	1.94–16.12
	No	13 (14.6%)	76 (85.4%)	89	1			
High depersonalization	Yes	10 (76.9%)	3 (23.1%)	13	12.32*	3.13–48.48	3.33	0.67–16.47
	No	23 (21.3%)	85 (78.7%)	108	1			
Low personal accomplishment	Yes	15 (51.7%)	14 (48.3%)	29	4.40*	1.81–10.75	2.84*	1.02–7.94
	No	18 (19.6%)	74 (80.4%)	92	1			

*Crude OR* crude odds ratio

* Significant at p < 0.05

### Relationships between sociodemographic factors and burnout syndrome

Several sociodemographic and educational factors were found to have an impact on whether or not the respondents experienced burnout (Table 4). Of all the factors tested, which include age, sex, marital status, raising children, and current level in residency, none had a statistically significant impact on the prevalence of burnout. Even though not found to be statistically significant, respondents who were currently alone based on their marital status (single or divorced) were more likely than their married counterparts to experience burnout (COR: 1.34, 95% CI 0.59–3.03). Similarly, those who were in residency years 1 and 2 were more likely than those in years 3 and 4 to have burnout (COR: 1.56, 95% CI 0.69–3.49; AOR: 1.52, 95% CI 0.60–3.81). Respondents between the ages of 24 and 28 years, males, and currently raising children were less likely to have burnout (p > 0.05). Beyond these factors, however, respondents who had received mental health help in the preceding 2 years before the study were 6.59 times significantly more likely on the Crude odds ratio scale (95% CI 2.60–16.70) to experience burnout than those who have not (p < 0.05).

**Table 4:** Relationship between burnout and socio-demographic/educational factors (n = 121)

Variables	Values	Burnout (n, %)		Total	Crude OR		Adjusted OR	
		Yes (n = 33)	No (n = 88)		OR	95% CI	OR	95% CI
Age	24–28 years	22 (26.8%)	60 (73.2%)	82	0.93	0.40–2.19	0.82	0.29–2.30
	≥ 29 years	11 (28.2%)	28 (71.8%)	39	1			
Sex	Male	15 (21.4%)	55 (78.6%)	70	0.50	0.22–1.12	0.61	0.25–1.53
	Female	18 (35.3%)	33 (64.7%)	51	1			
Marital status	Single/divorced	20 (29.9%)	47 (70.1%)	67	1.34	0.59–3.03	0.97	0.34–2.77
	Married	13 (24.1%)	41 (75.9%)	54	1			
Raising children	Yes	5 (17.2%)	24 (82.8%)	29	0.48	0.17–1.38	0.54	0.13–2.16
	No	28 (30.4%)	64 (69.6%)	92	1			
Current level	R1 – R2	19 (31.7%)	41 (68.3%)	60	1.56	0.69–3.49	1.52	0.60–3.81
	R3 – R4	14 (23.0%)	47 (77.0%)	61	1			
Got mental help	Yes	16 (59.3%)	11 (40.7%)	27	6.59*	2.60–16.70	5.80*	2.19–15.35
	No	17 (18.1%)	77 (81.9%)	94	1			

*Crude OR* crude odds ratio

* Significant at p < 0.05

### Relationships between sociodemographic factors and depressive symptoms

With regards to factors influencing depressive symptoms, age, sex, and current level in residency were factors found to have a statistically significant influence on whether or not the respondents were depressed (Table 5). Respondents who were between the ages of 24 and 28 were less likely than those 29 years or more to have depressive symptoms (COR: 0.65, 95% CI 0.28–1.49; AOR: 0.29, 95% CI 0.09–0.91, p < 0.05). Similarly, males were less likely than females to have depressive symptoms (COR: 0.20, 95% CI 0.09–0.48; AOR: 0.15, 95% CI 0.05–0.42, p < 0.05). Being in the first and second years of residency significantly increases the chances of having depressive symptoms as the respondents’ data shows (COR: 2.20, 95% CI 0.96–5.02; AOR: 3.29, 95% CI 1.16–9.34, p < 0.05). Residents who have received any mental health help in the last 2 years were significantly more likely than those who have not to have depressive symptoms (COR: 5.28, 95% CI 2.11–13.20; AOR: 3.97, 95% CI 1.38–11.38, p < 0.05). Even though not statistically significant, respondents who were alone, because they were single or divorced were more likely than their married counterparts to have depressive symptoms (COR: 1.34, 95% CI 0.59–3.03; AOR: 1.10, 95% CI 0.36–3.37).

**Table 5:** Relationship between depressive symptoms and socio-demographic/educational factors (n = 121)

Variables	Values	Depressive symptoms (n, %)		Total	Crude OR		Adjusted OR	
		Yes (n = 33)	No (n = 88)		OR	95% CI	OR	95% CI
Age	24–28 years	20 (24.4%)	62 (75.6%)	82	0.65	0.28–1.49	0.29*	0.09–0.91
	≥ 29 years	13 (33.3%)	26 (66.7%)	39	1			
Sex	Male	10 (14.3%)	60 (85.7%)	70	0.20*	0.09–0.48	0.15*	0.05–0.42
	Female	23 (45.1%)	28 (54.9%)	51	1			
Marital status	Single/divorced	20 (29.9%)	47 (70.1%)	67	1.34	0.59–3.03	1.10	0.36–3.37
	Married	13 (24.1%)	41 (75.9%)	54	1			
Raising children	Yes	5 (17.2%)	24 (82.8%)	29	0.48	0.17–1.38	0.50	0.12–2.15
	No	28 (30.4%)	64 (69.6%)	92	1			
Current level	R1 – R2	21 (35.0%)	39 (65.0%)	60	2.20	0.96–5.02	3.29*	1.16–9.34
	R3 – R4	12 (19.7%)	49 (80.3%)	61	1			
Got mental help	Yes	15 (55.6%)	12 (44.4%)	27	5.28*	2.11–13.20	3.97*	1.38–11.38
	No	18 (19.1%)	76 (80.9%)	94				

*Crude OR* crude odds ratio

* Significant at p < 0.05

## Discussion

This study aims to assess the prevalence of burnout, depressive symptoms, and their correlation during the COVID-19 pandemic among psychiatry residents in Saud Arabia. Our study revealed that about 27.3% of the participants were suffering from burnout. This finding seems to be lower than the results of the study by Jovanović and colleagues, who reported the prevalence of burnout among psychiatric trainees in 22 countries to be 36.7% [11]. However, our finding is within the range of burnout rates reported by residents from different medical specialties (13% to 80%) [22]. The lower prevalence in our study could be attributed to the reduction in all hospital activity and resident duties in the early days of the pandemic. However, psychiatry residents are among the entire health-care system that is thought to be affected during the pandemic. Multiple sources are thought to increase burnout among the study population during the pandemic. Some of these sources are the new policies and rules, stress among staff, and the fact that infected individuals could be encountered in the workplace at any time [23]. Factors not related directly to the pandemic could also contribute to burnout among the study population, such as dealing with suicidal and homicidal patients, difficulty separating one’s personal life from professional life, and dealing with sensitive and emotional patients [24]. In addition, psychiatrists’ personality traits may make them more vulnerable to burnout as they tend to internalize their stressful experiences [25].

Regarding depression, our result showed that 27.3% of the participants were suffering from depressive symptoms. This result is in line with the result of a rapid systematic review conducted by Ricci-Cabello and colleagues. They reported that the pool prevalence of depression among health care workers during a viral epidemic outbreak was 38% [10]. Our study also aimed to analyze the complex associations between depressive symptoms and burnout among the study population. We found a significant relationship between burnout and depressive symptoms. It is still debatable whether burnout syndrome and depressive symptoms arise from the same situation, or are two different conditions [26]. There was a statistically significant positive correlation between all three burnout subscales (EE, DP, and PA) and depressive symptoms. This strong correlation highlights the importance of early recognition of burnout symptoms, where it is significantly predictive of depressive symptoms.

Burnout among psychiatry residents was associated with several sociodemographic factors that have an impact on whether the participants experienced burnout or not. In this study, the sociodemographic data, including age, sex, marital status, and raising children, had no statistically significant impact on the prevalence of burnout. Similar findings were reported in a systematic review by Chan MK et al. [14]. They concluded that the data regarding the age and gender of participants were inconsistent in the rates of burnout.

However, in this study, we found that being alone (single, divorced) and in junior years of training (residence years one and two) were associated with experiencing burnout more than counterparts. This finding is consistent with many studies conducted among psychiatry residents included in a systematic review by Chan [14]. For example, Kealy and colleagues [27] reported that burnout rates in program year (PGY)-2 and PGY-3 residents ranged from 27 to 31% compared with 16% to 18% in PGY-4 and PGY-5 residents. Moreover, a study conducted among medical residents during the H1N1 outbreak in Mexico reported that younger age was a risk factor for burnout [28]. In our study, increased reporting of burnout during the first 2 years of training can be attributed to many factors, which could be related to the pressure of fast skill attainment in assessing, diagnosing, and managing patients while experiencing the circumstances surrounding dealing with psychiatric patients with COVID-19 in emergency, inpatient, and outpatient units.

In addition, respondents currently raising children were less likely to have burnout, as was observed in previous studies [29]^11^. This finding is also in agreement with a study conducted among emergency department nurses during the Middle East respiratory syndrome coronavirus in Korea [30].

Relationships between sociodemographic factors and depressive symptoms were mostly consistent with the results of previous studies [15]. Similar to the result shown in our study, females were reported to have more depression than males among psychiatry residents and the general population [15, 31]. In addition, a recent study in China during the COVID-19 pandemic showed a similar result, of more females with depression than males among health care workers [32]. Moreover, residents in their first and second years of training had an increased chance of having depressive symptoms compared to other years, and this finding is consistent with findings from another study [33].

Respondents who had received mental health help in the preceding 2 years before the study were 6.59 times significantly more likely to experience burnout and depressive symptoms than those who had not. An explanation for this finding is that respondents who received mental health help, whether due to having a mental illness or facing difficulty with stressors, are at higher risk of developing burnout. This emphasizes the importance of intrinsic factors related to personality traits that make them prone to internalize their stressful experience as well as deficient in coping skills to deal with the current pandemic [34].

Much attention has been paid to the frontline health care workers, although that all health care providers are affected by COVID-19, especially those in training. As highlighted by the World Health Organization on May 14, 2020, there is a need to urgently increase investment in mental health services or risk a massive increase in mental health conditions in the coming months [35]. Under these circumstances, mental health workers will be under more pressure and could be more prone to burnout in these coming months. Hence, the need to construct a plan to reduce this risk is more important than ever by conducting future studies and developing preventative strategies and effective treatment programs to prevent burnout and promote wellness.

When researching burnout, it may be difficult to decide whether to report the results separately for each dimension of burnout or whether to combine the dimensions. While it is preferable to treat burnout as a multidimensional construct for theoretical purposes, it is often more convenient for researchers to treat burnout as a unidimensional variable.[36].

One limitation of our study design that it cannot determine the impact of a pandemic. Other limitations include that the focus of the research was limited to a subgroup population in one country; therefore, it is important to expand the scope of participants to compare it with different cases in different countries. Moreover, at the time of data collection, the pandemic was at its early stages in Saudi Arabia, which might not represent the current burnout and depressive symptoms. In addition, factors such as weekly working hours and the type of care provided by residents, being in contact with COVID-19 patients, personality traits, coping plans, and job attitude could be examined as other factors influencing burnout and depression. Finally, follow-up studies are needed to assess progression or even a potential rebound effect of psychological manifestations once the imminent threat of COVID-19 subsides.

## Conclusions

In conclusion, during pandemics, the role of psychiatrists as a source of psychological support and reassurance to the public and frontline workers is indispensable and vital, and their mental wellbeing is critical to meet these needs. In this study, the prevalence of burnout and depressive symptoms during the COVID-19 outbreak was assessed among psychiatry residents in Saudi Arabia. Burnout symptoms were found in 27.3%, and another 27.3% reported having depression symptoms. In addition, 16.5% reported having both burnout and depressive symptoms, with a significant relationship between the two conditions. Participants who were in the first 2 years of training and had a history of receiving mental health in the past 2 years are at higher risk of developing burnout and depressive symptoms. During pandemics, as highlighted by the World Health Organization, there is a need to urgently increase investment in mental health. Hence, the need to construct a plan to reduce this risk of burnout and depression is more important than ever by conducting future studies and developing preventative strategies and effective treatment programs to prevent burnout and promote wellness.

## Data Availability

The datasets used and/or analyzed during the current study are available from the corresponding author on reasonable request.

## Abbreviations

COVID-19: Coronavirus disease of 2019
MBI-HSS: Maslach Burnout Inventory (Human Services Survey)
PHQ-9: Patient’s Health Questionnaire
EE: Emotional exhaustion
DP: Depersonalization
PA: Personal accomplishment
PGY: Program year
